# Adjuvant psychotherapy in early-stage bipolar disorder: A protocol for systematic review and meta-analysis

**DOI:** 10.1097/MD.0000000000025443

**Published:** 2021-04-09

**Authors:** Qianfang Chen, Yuanyue Zhou, Huifen Lv, Caie Ma

**Affiliations:** a Department of neurology; b Department of Children's psychology; c Department of psychiatry, Hangzhou Seventh People's Hospital, Zhejiang, China.

**Keywords:** bipolar disorder, meta-analysis, pharmacotherapy, psychotherapy

## Abstract

**Objective:**

There is no systematic review or meta-analysis to evaluate the efficacy of adjuvant psychotherapy in early-stage bipolar disorder. Therefore, the goal of this meta-analysis is to examine the evidence supporting psychotherapy as an efficacious approach to treating bipolar disorder.

**Methods:**

Seven electronic databases including Web of Science, Embase, PubMed, Wanfang Data, Scopus, Science Direct, Cochrane Library were searched in March 2021 by two independent reviewers. Data extraction was performed independently, and any conflict was resolved before final analysis. Only randomized clinical trials were included in this study. The trial entails 1 primary outcome measure (relapse) and several secondary outcome measures: time to relapse, relapse rate, days missed at work/school (record, interview), and social functioning level. The risk of bias assessment of the included studies was performed by 2 authors independently using the tool recommended in the Cochrane Handbook for Systematic Reviews of Interventions.

**Results:**

We hypothesized that combined psychotherapy and pharmacological interventions would be superior to pharmacological interventions alone regarding the time to relapse into a manic or depressive episode.

**Conclusion:**

This study expects to provide credible and scientific clinical evidence for the efficacy and safety of combined psychotherapy and pharmacological interventions in the treatment of bipolar disorder.

**OSF registration number:**

10.17605/OSF.IO/ZGS6W

## 1 Introduction

Bipolar disorder is a chronic affective condition characterized by episodes of mania and depression.^[1,2]^ Two major types of bipolar disorder have been defined: bipolar I and bipolar II.^[3]^ Manic episodes are the dominant feature of bipolar I; however, depressive episodes are also common. In bipolar II, manic symptoms have lower intensity and duration (hypomania), while depression is more pronounced. In addition, patients with bipolar disorder may experience episodes that combine the features of both mania and depression.^[4,5]^ A large cross-sectional survey of 11 countries found the overall lifetime prevalence of bipolar spectrum disorders was 2.4%, with a prevalence of 0.6% for bipolar type I and 0.4% for bipolar type II.^[6]^ The onset age of bipolar disorders is typically during late adolescence and early adulthood. This is a very sensitive phase for educational, professional, and social development. In addition, this is a critical time in the developmental lifespan characterized by the establishment of one's personality and often experimentation with oppositional attitudes, chaotic social and sleeping rhythms, and drug use.^[7]^

Over the past few decades, there has been increasing attention to the development of bipolar disorder-specific psychotherapies.^[8]^ In part, this resurgence is related to disappointingly low remission and recovery rates, despite more pharmacotherapy options and growing efforts to personalize treatment. Pharmacological interventions are essential to the management of bipolar disorder, needed for all except a subset of individuals with bipolar disorder type II, for whom psychotherapy may be an adequate monotherapy.^[9,10]^ However, even when pharmacotherapy follows best-practice guidelines, it is effective in reducing only some symptoms, some relapses, and some suicides.^[11,12]^ A comprehensive treatment approach that includes pharmacotherapy and an evidence-based psychotherapy may provide the strongest foundation for increasing self-efficacy, reducing symptoms and recurrences, and restoring functioning and quality of life.

However, there is no systematic review or meta-analysis to evaluate the efficacy of adjuvant psychotherapy in early-stage bipolar disorder. Therefore, the goal of this meta-analysis is to examine the evidence supporting psychotherapy as an efficacious approach to treating bipolar disorder.

## 2 Materials and methods

### 2.1 Protocol registration

The prospective registration has been approved by the Open Science Framework (OSF) registries (https://osf.io/zgs6w), and the registration number is 10.17605/OSF.IO/ZGS6W. The protocol was written following the Preferred Reporting Items for Systematic Reviews and Meta-Analyses Protocols statement guidelines.

### 2.2 Selection of studies

**Figure 1:**
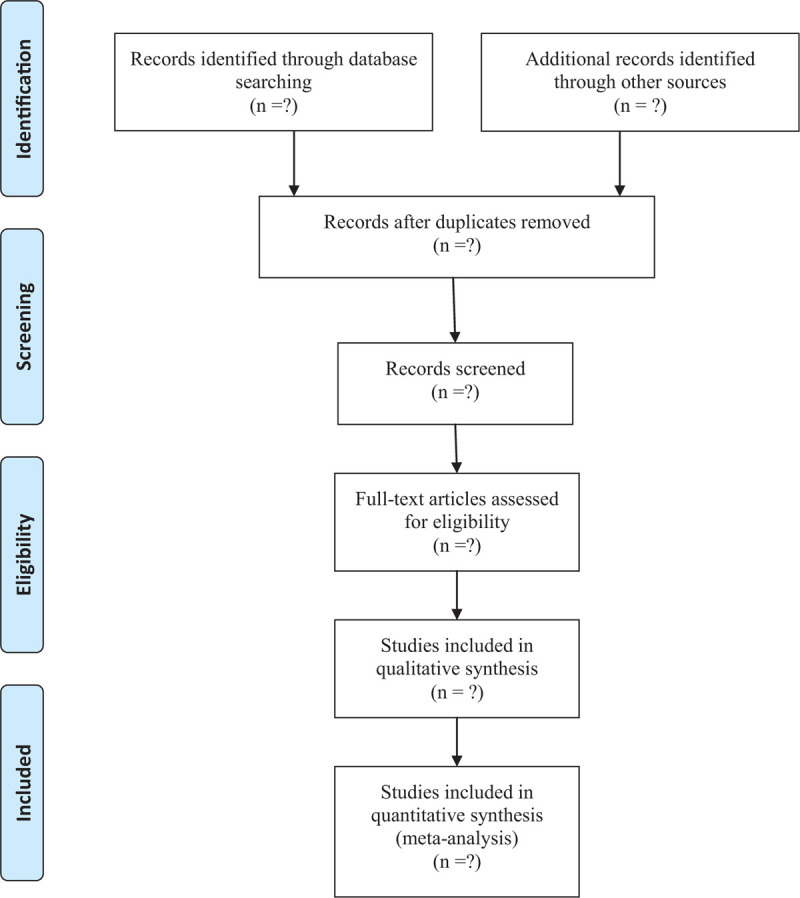
Flow diagram of study selection.

Seven electronic databases including Web of Science, Embase, PubMed, Wanfang Data, Scopus, Science Direct, Cochrane Library were searched in March 2021 by two independent reviewers. The established search strategy for PubMed was displayed in Table 1. The reference lists of the included studies were also checked for additional studies that were not identified with the database search. There was no restriction in the dates of publication or language in the search. No ethical approval was required in our study because all analyses were based on aggregate data from previously published studies (Fig. 1).

**Table 1:** Search strategy of PubMed.

Number	Search items
Mesh term #1	((bipolar disorder) OR (mental disorders) OR (depression) OR (mania) OR (affective disorder) OR (affective insanity) OR (pendulum disease) OR (spirit system disease) OR (mental illness) OR (bipolar spectrum disorders)
Mesh term #2	((psychotherapy) OR (cognitive therapy) OR (mental intervention) OR (psychological intervention) OR (behavior therapy) OR (Morita therapy) OR (psychological counseling))
Mesh term #3	((clinical trials) OR (randomized controlled trials))
#1 AND #2 AND #3	

Mesh = medical subject heading.

### 2.3 Inclusion and exclusion criteria

Study included in this systematic review and meta-analysis had to meet all of the following inclusion criteria in the PICOS order:

(1) participants: patients with bipolar disorder;
(2) intervention: patients received combined pharmacological interventions and psychotherapy;
(3) comparator: patients received pharmacological interventions;
(4) outcomes: the trial entails one primary outcome measure (relapse) and several secondary outcome measures: time to relapse, relapse rate, days missed at work/school (record, interview), and social functioning level;
(5) study design: randomized controlled trials.

The exclusion criteria were as follows:

(1) studies which did not assessed the above outcomes;
(2) lack of a control group;
(3) studies with the following types: case reports, comments or letters, biochemical trials, protocols, conference abstracts, reviews, and retrospective studies or prospective non-randomized studies.

### 2.4 Study selection

Articles were exported to EndNote, and duplicates removed. Two independent authors screened the titles and abstracts of potentially relevant studies to determine their eligibility based on the criteria. Disagreements were resolved through a discussion with a third review author.

### 2.5 Data extraction

Data were extracted by review of each study for population, mean age, gender, follow-up duration, study design, publishing date, intervention methods, and outcomes assessment. The two reviewers created a study-specific speadsheet in Excel (Microsoft Corp., Redmond) for data collection. Data extraction was performed independently, and any conflict was resolved before final analysis. Any disagreements between the two reviewers were discussed and, if necessary, the third author was referred to for arbitration. If the data were missing or could not be extracted directly, authors were contacted by email. Otherwise, we calculated them with the guideline of Cochrane Handbook for Systematic Reviews of Interventions 5.1.0. If necessary, we would abandon the extraction of incomplete data.

### 2.6 Quality assessment

The risk of bias assessment of the included studies was performed by two authors independently using the tool recommended in the Cochrane Handbook for Systematic Reviews of Interventions (version 5.1.0).^[13]^ This tool included seven aspects which were sequence generation (selection bias), allocation sequence concealment (selection bias), blinding of participants and personnel (performance bias), blinding of outcome assessment (detection bias), incomplete outcome data (attrition bias), selective outcome reporting (reporting bias) and other bias (baseline balance and fund). Additionally, each of the aspects was ranked low risk of bias, high risk of bias, and unclear risk of bias. The evidence grade was assessed using the guidelines of the Grading of Recommendations, Assessment, Development, and Evaluation working group including the following items: risk of bias, inconsistency, indirectness, imprecision and publication bias.^[14]^ The recommendation level of evidence was classified into the following categories:

(1) high, which means that further research is unlikely to change confidence in the effect estimate;
(2) moderate, which means that further research is likely to significantly change confidence in the effect estimate but may change the estimate;
(3) low, which means that further research is likely to significantly change confidence in the effect estimate and to change the estimate; and
(4) very low, which means that any effect estimate is uncertain.

Grading of Recommendations Assessment, Development and Evaluation pro Version 3.6 software is used for the evidence synthesis.

### 2.7 Statistical analysis

Data analysis was performed with Review Manager Software (RevMan Version 5.4, The Cochrane Collaboration, Copenhagen, Denmark). As outcomes which assessed pain intensity might be reported on different scores, we used the standardized mean difference with a 95% confidence interval to assess for these outcomes. A *P* value < .05 was considered statistically significant. All outcomes were pooled on random-effect model. The statistical heterogeneity was assessed by using the Cochrane *Q* test and *I*^2^ statistic. The low, moderate, and high heterogeneity were assigned to *I*^2^ values of 0% to 25%, 26% to 74%, and above 75%. A meta-analysis was conducted when 4 or more trials reported an outcome of interest. A sensitivity analysis was planned by different follow-up periods. Begg's funnel plot was used to assess publication bias. If publication bias exists, the Begg's funnel plot is asymmetric.

## 3 Discussion

Although pharmacotherapy is the mainstay of treatment for bipolar disorder, medication offers only partial relief for patients. Treatment with pharmacologic interventions alone is associated with disappointingly low rates of remission, high rates of recurrence, residual symptoms, and psychosocial impairment. Bipolar-specific therapy is increasingly recommended as an essential component of illness management. To the best of our knowledge, this is the first meta-analysis from randomized controlled trials to assess the efficacy of adjuvant psychotherapy for the treatment of bipolar disorder. We hypothesized that combined psychotherapy and pharmacological interventions would be superior to pharmacological interventions alone regarding the time to relapse into a manic or depressive episode. The second aim of this study is to measure the effect of our interventions on other outcome parameters (social functioning) and further, to identify clinical and neurobiological predictors for successful psychotherapeutic interventions in bipolar disorders. The review will add to the existing literature by showing compelling evidence and improved guidance in clinic settings.

Caie Ma designs the protocol. Yuanyue Zhou and Huifen Lv perform the data collection. Qianfang Chen writes the manuscript. All of the authors approved the submission.

## Author contributions

**Conceptualization:** Yuanyue Zhou.

**Data curation:** Huifen Lv.

**Formal analysis:** Huifen Lv.

**Funding acquisition:** Caie Ma.

**Writing – original draft:** Qianfang Chen.
